# Supplementary material for: Interneuron FGF13 regulates seizure susceptibility via a sodium channel-independent mechanism
Source: eLife. 2025 Jan 8;13:RP98661. doi: 10.7554/eLife.98661 (PMC11709433; doi:10.7554/eLife.98661)
Supplement: Figure 1—source data 1. [file elife-98661-fig1-data1.zip › Figure 1B source data/Figure 1B-Source Data.pdf]

Figure 1B-Source Data

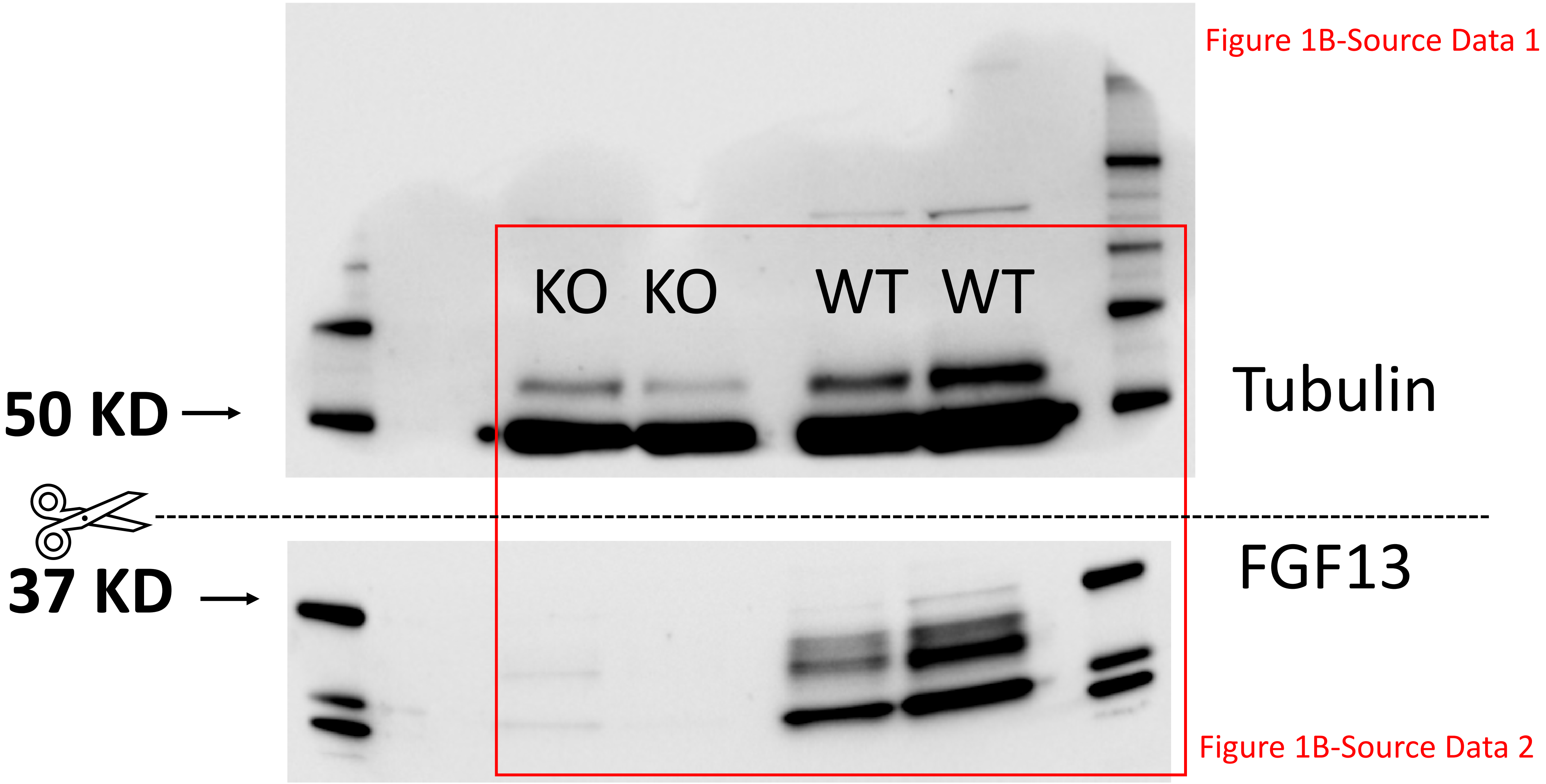

Gel cut where indicated, probed separately for tubulin (top) and FGF13 (bottom).
Image flipped horizontally
