# Supplementary figures and images for: Interneuron FGF13 regulates seizure susceptibility via a sodium channel-independent mechanism
Source: eLife. 2025 Jan 8;13:RP98661. doi: 10.7554/eLife.98661 (PMC11709433; doi:10.7554/eLife.98661)

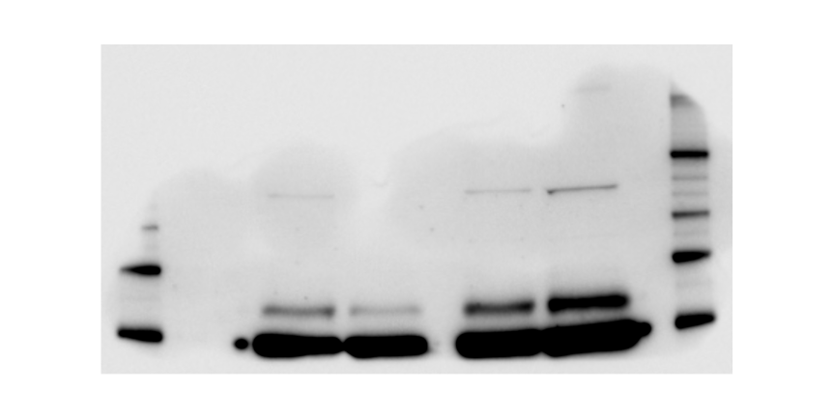

Supplement: Figure 1—source data 1. [file elife-98661-fig1-data1.zip › Figure 1B source data/Figure 1B-Source Data 1.tif]

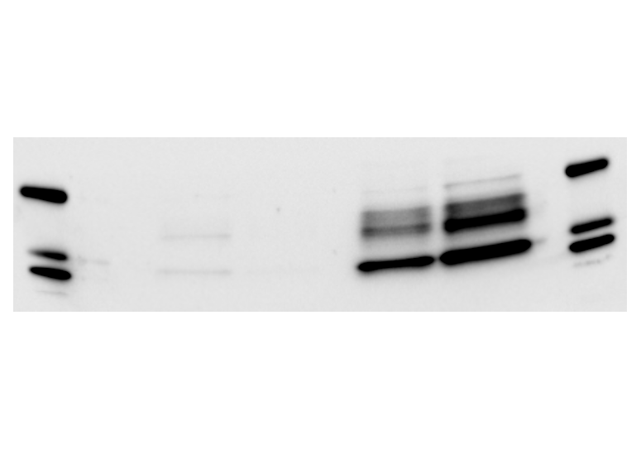

Supplement: Figure 1—source data 1. [file elife-98661-fig1-data1.zip › Figure 1B source data/Figure 1B-Source Data 2.tif]

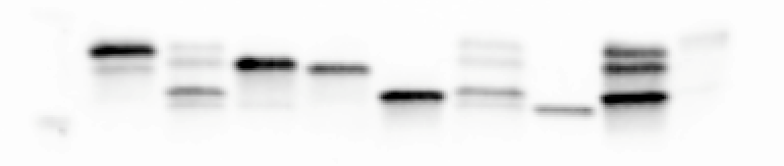

Supplement: Figure 1—figure supplement 1—source data 1. [file elife-98661-fig1-figsupp1-data1.zip › Figure 1-figure supplement 1B source data/Figure 1-figure supplement 1B-source data 1.tif]

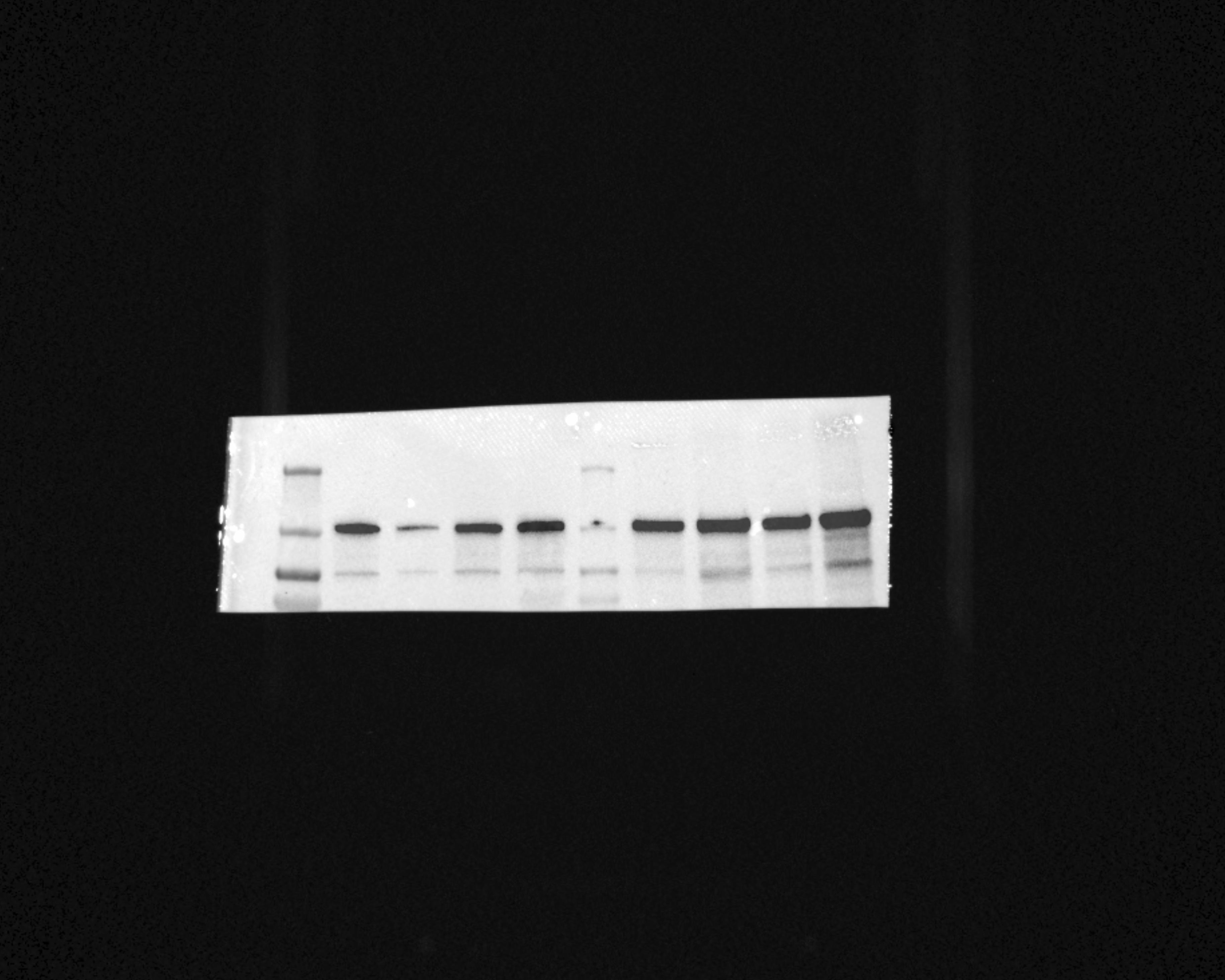

Supplement: Figure 1—figure supplement 1—source data 1. [file elife-98661-fig1-figsupp1-data1.zip › Figure 1-figure supplement 1B source data/Figure 1-figure supplement 1B-source data 2.tif]

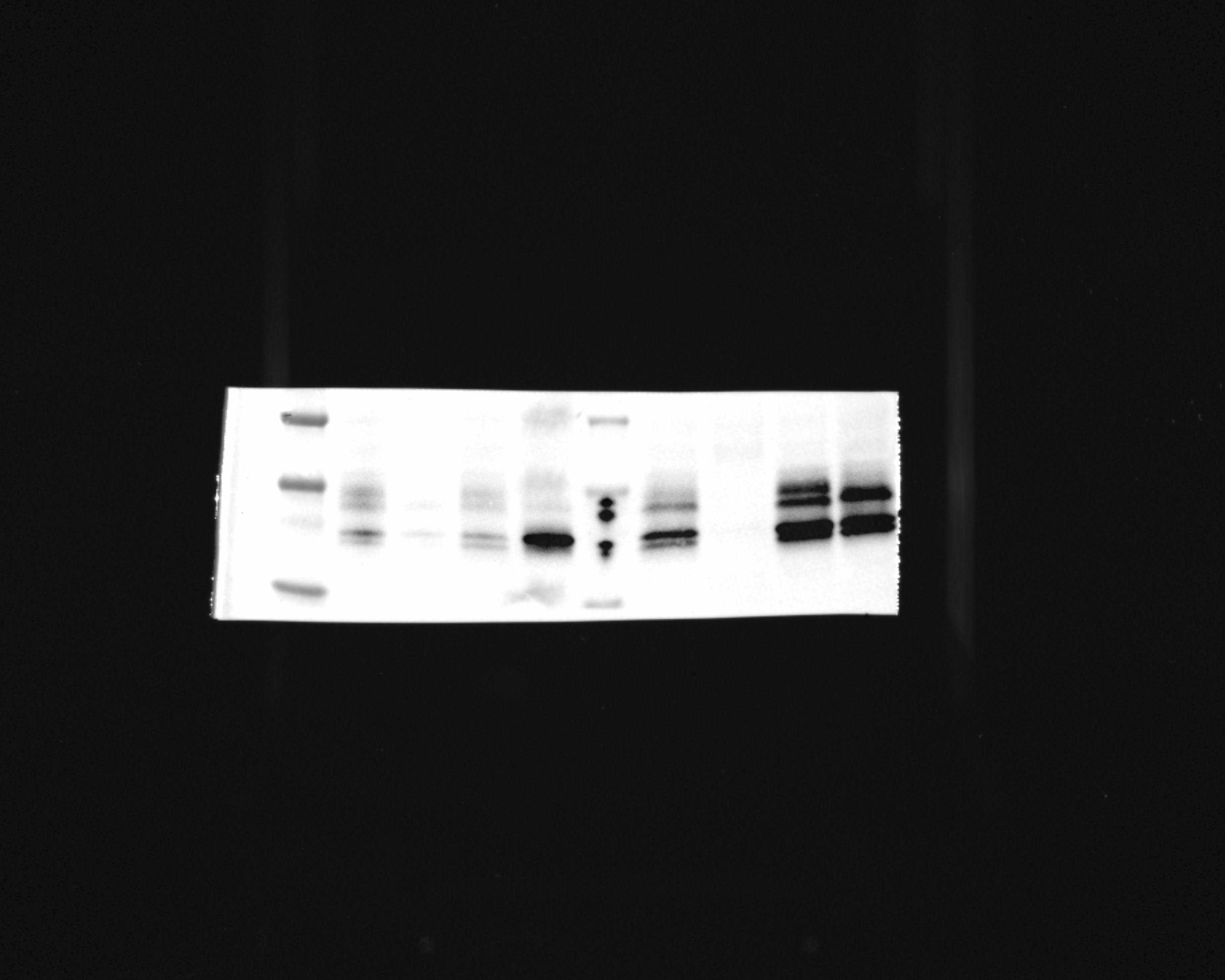

Supplement: Figure 1—figure supplement 1—source data 1. [file elife-98661-fig1-figsupp1-data1.zip › Figure 1-figure supplement 1B source data/Figure 1-figure supplement 1B-source data 3.tif]

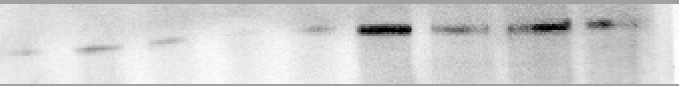

Supplement: Figure 2—source data 1. [file elife-98661-fig2-data1.zip › Figure 2A source data/Figure 2A-source data 1.tif]

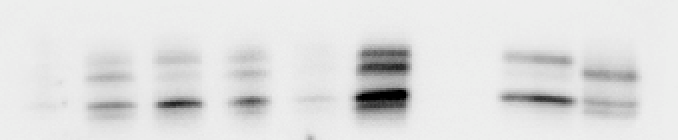

Supplement: Figure 2—source data 1. [file elife-98661-fig2-data1.zip › Figure 2A source data/Figure 2A-source data 2.tif]

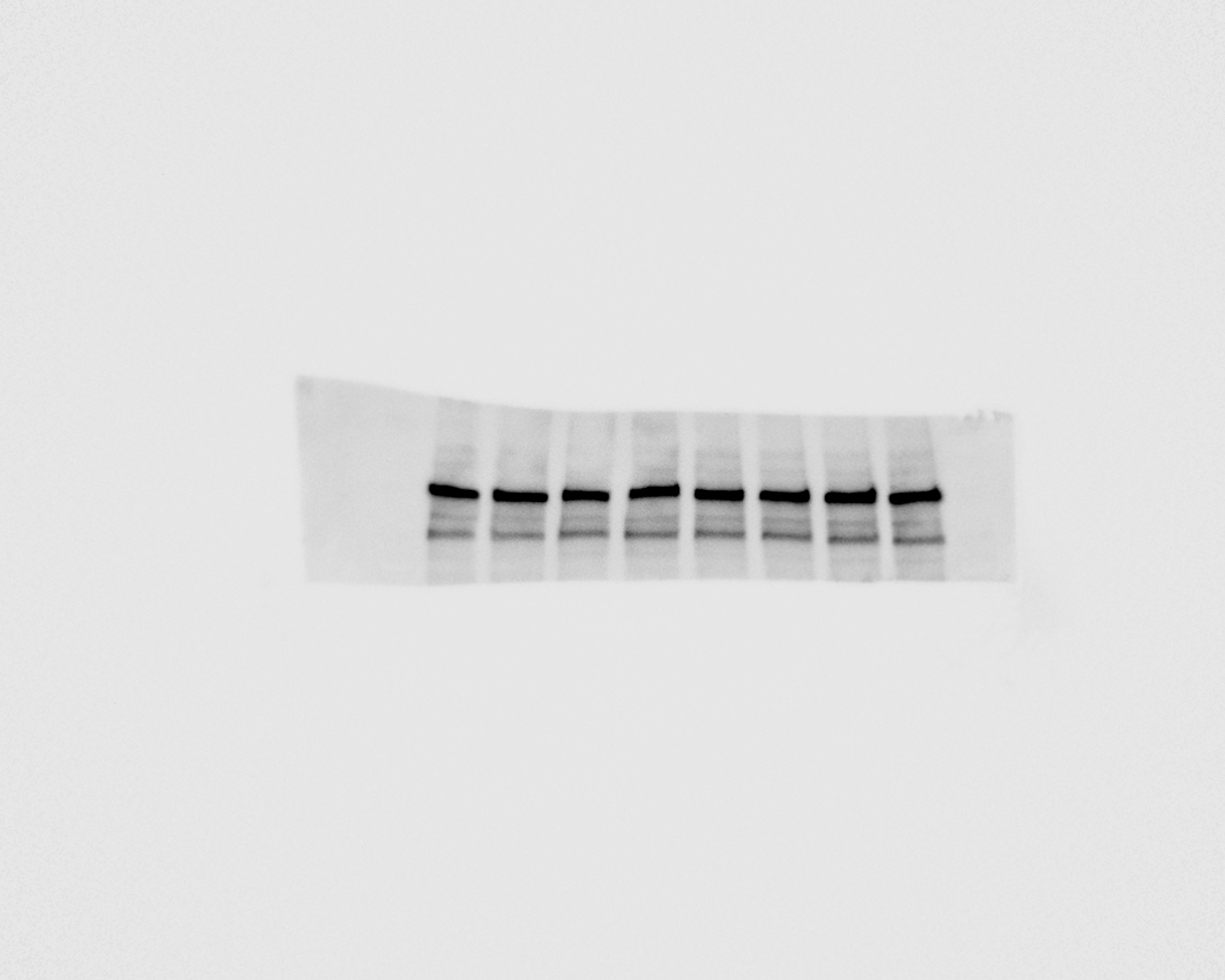

Supplement: Figure 2—figure supplement 1—source data 1. [file elife-98661-fig2-figsupp1-data1.zip › Figure 2-figure supplement 1 source data/Figure 2-figure supplement 1-source data 1(Chemiluminescence).jpg]

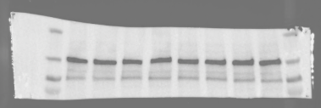

Supplement: Figure 2—figure supplement 1—source data 1. [file elife-98661-fig2-figsupp1-data1.zip › Figure 2-figure supplement 1 source data/Figure 2-figure supplement 1-source data 1.tif]

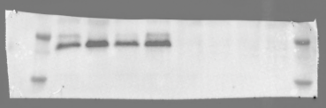

Supplement: Figure 2—figure supplement 1—source data 1. [file elife-98661-fig2-figsupp1-data1.zip › Figure 2-figure supplement 1 source data/Figure 2-figure supplement 1-source data 2.tif]

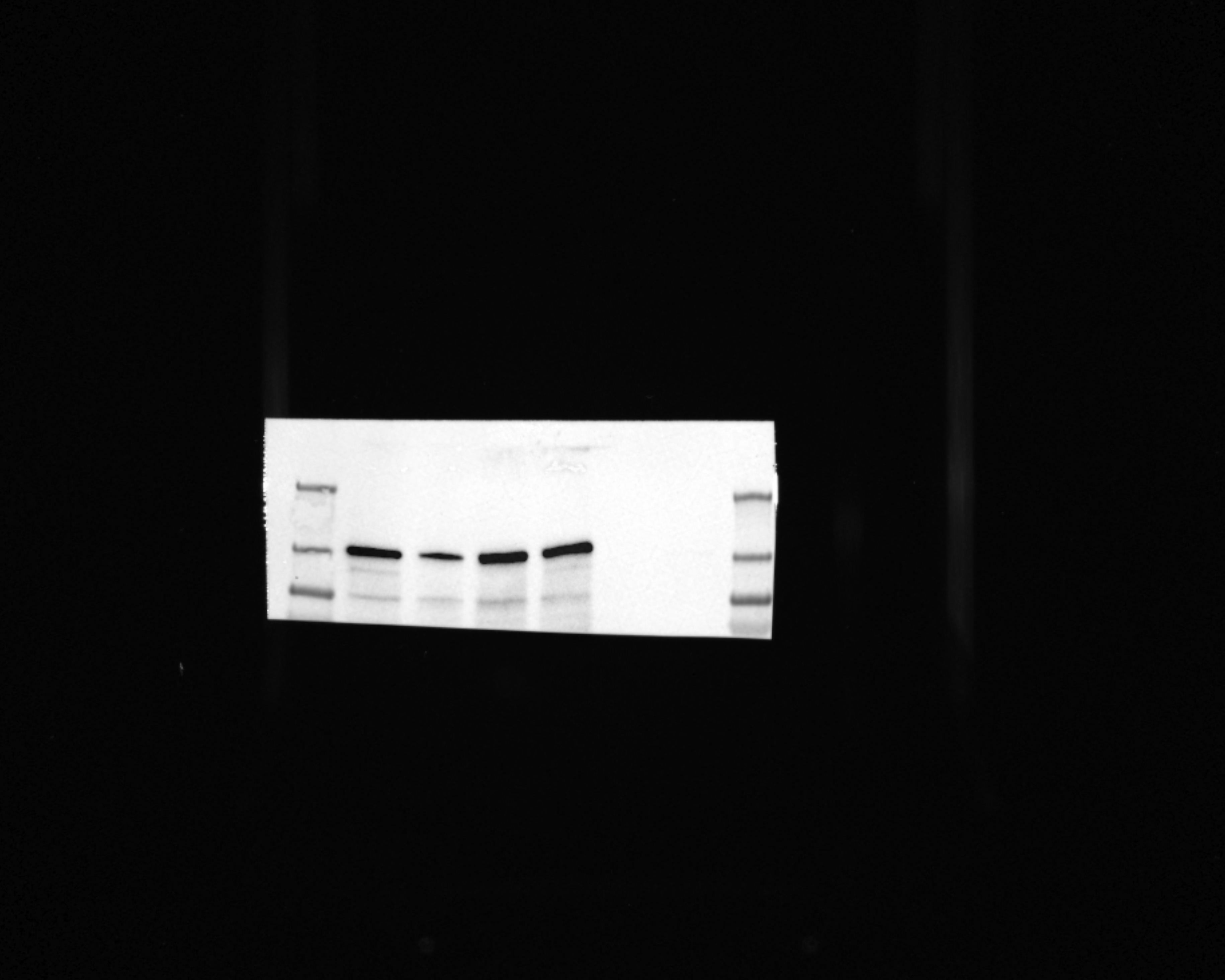

Supplement: Figure 3—source data 1. [file elife-98661-fig3-data1.zip › Figure 3C source data/Figure 3C-Source Data 1.tif]

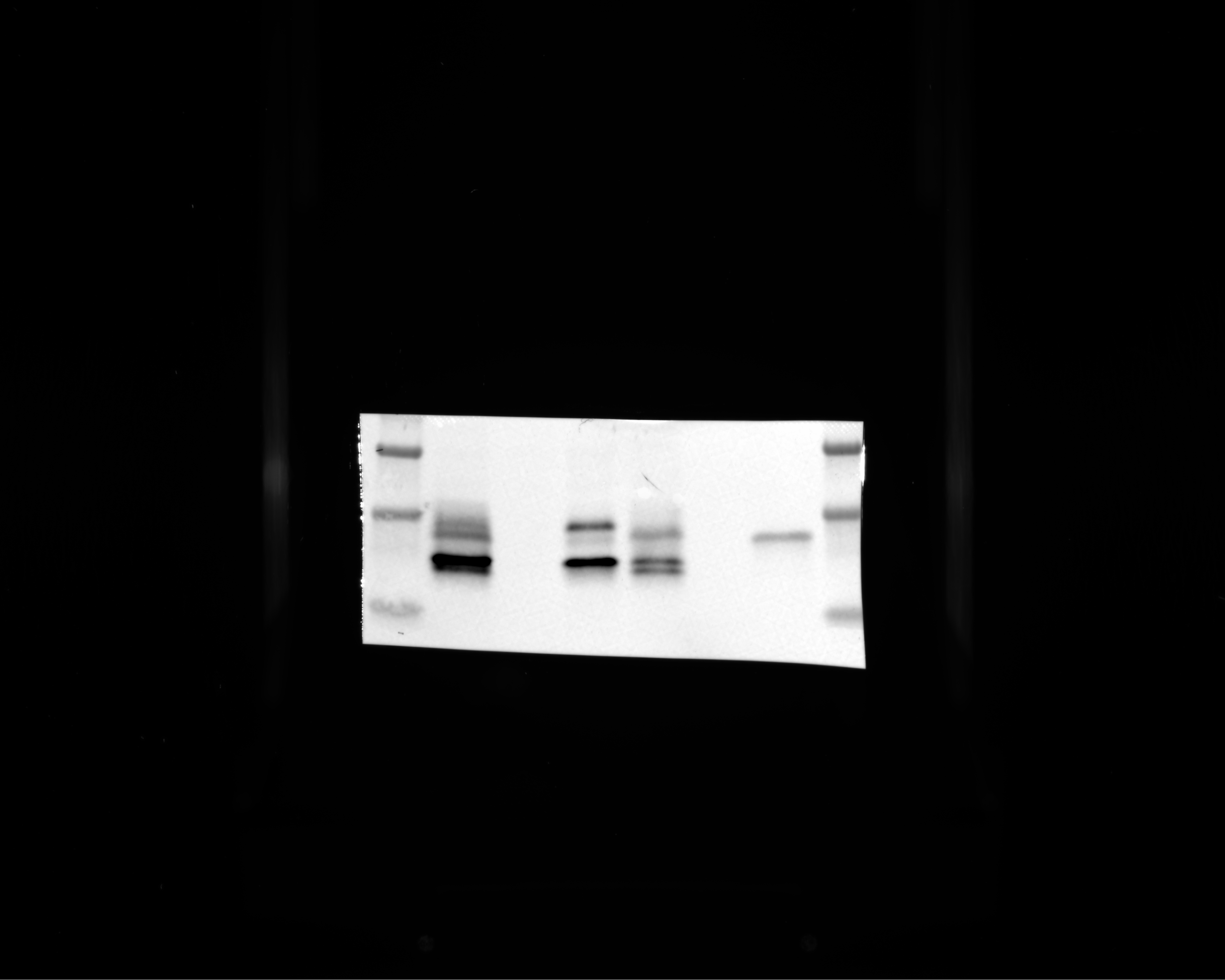

Supplement: Figure 3—source data 1. [file elife-98661-fig3-data1.zip › Figure 3C source data/Figure 3C-Source Data 2.tif]
