# Supplementary material for: Interneuron FGF13 regulates seizure susceptibility via a sodium channel-independent mechanism
Source: eLife. 2025 Jan 8;13:RP98661. doi: 10.7554/eLife.98661 (PMC11709433; doi:10.7554/eLife.98661)
Supplement: Figure 2—source data 1. [file elife-98661-fig2-data1.zip › Figure 2A source data/Figure 2A-source data.pdf]

Figure 2A-Source data

Imaged 5-19-2022
4-15% BioRad Tris-Glycine gel
200V for 40 min

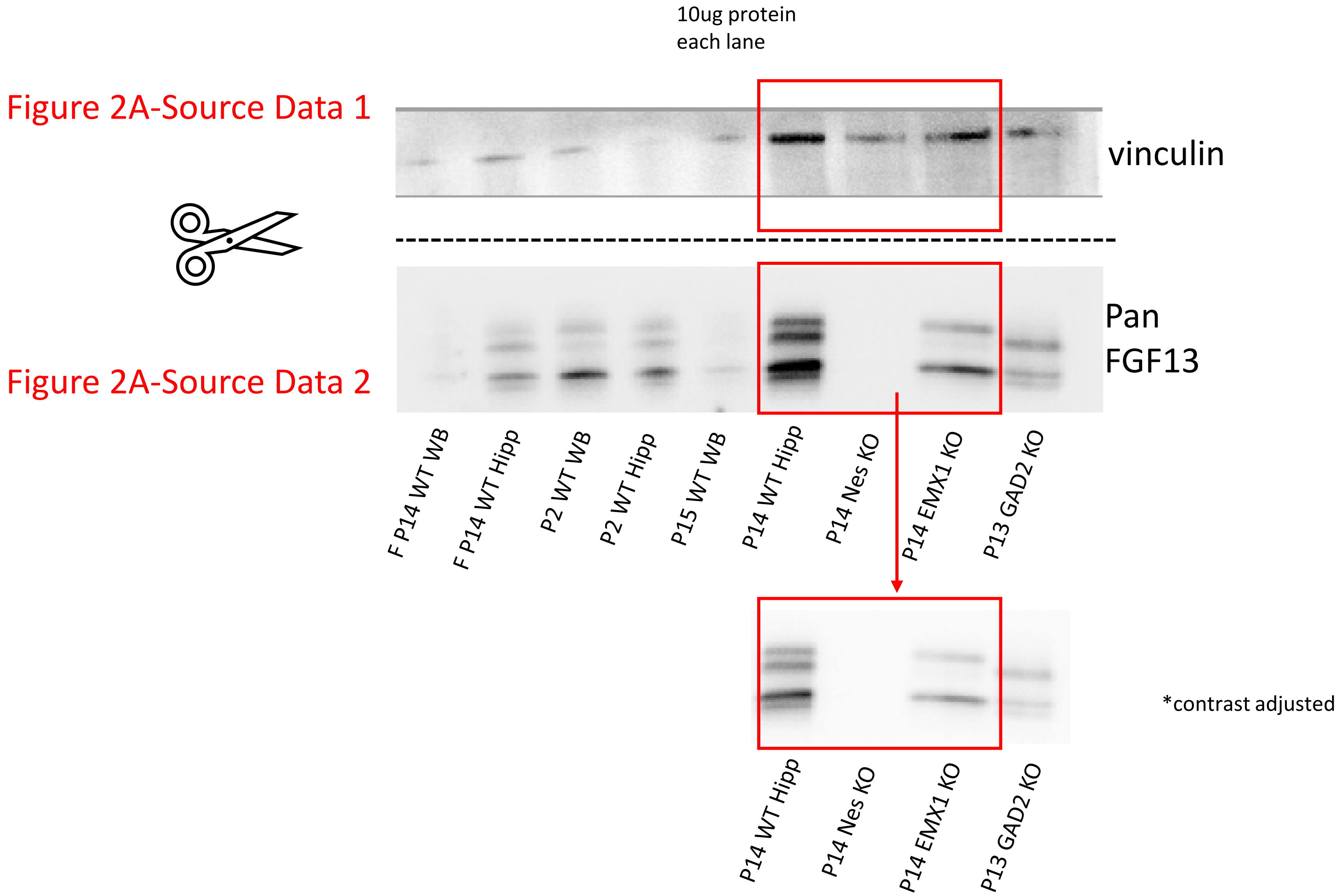

Gel cut where indicated, probed separately for vinculin (top) and FGF13 (bottom). Image contrast for FGF13 adjusted
