# Supplementary material for: Interneuron FGF13 regulates seizure susceptibility via a sodium channel-independent mechanism
Source: eLife. 2025 Jan 8;13:RP98661. doi: 10.7554/eLife.98661 (PMC11709433; doi:10.7554/eLife.98661)
Supplement: Figure 2—figure supplement 1—source data 1. [file elife-98661-fig2-figsupp1-data1.zip › Figure 2-figure supplement 1 source data/Figure 2-figure supplement 1-source data.pdf]

Figure 2-figure supplement 1 -Source data 1

Shows colorimetric and chemiluminescent signal

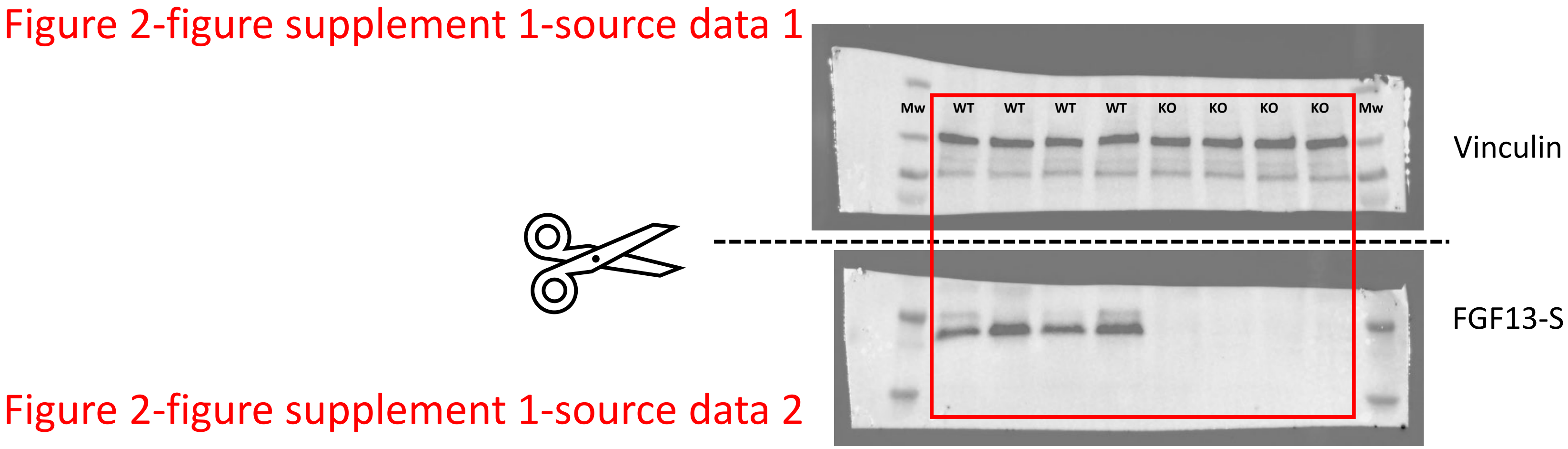

Gel cut where indicated, probed separately for vinculin (top) and FGF13 (bottom).

Figure 2-figure supplement 1 -Source data 1

Shows ONLY chemiluminescent signal

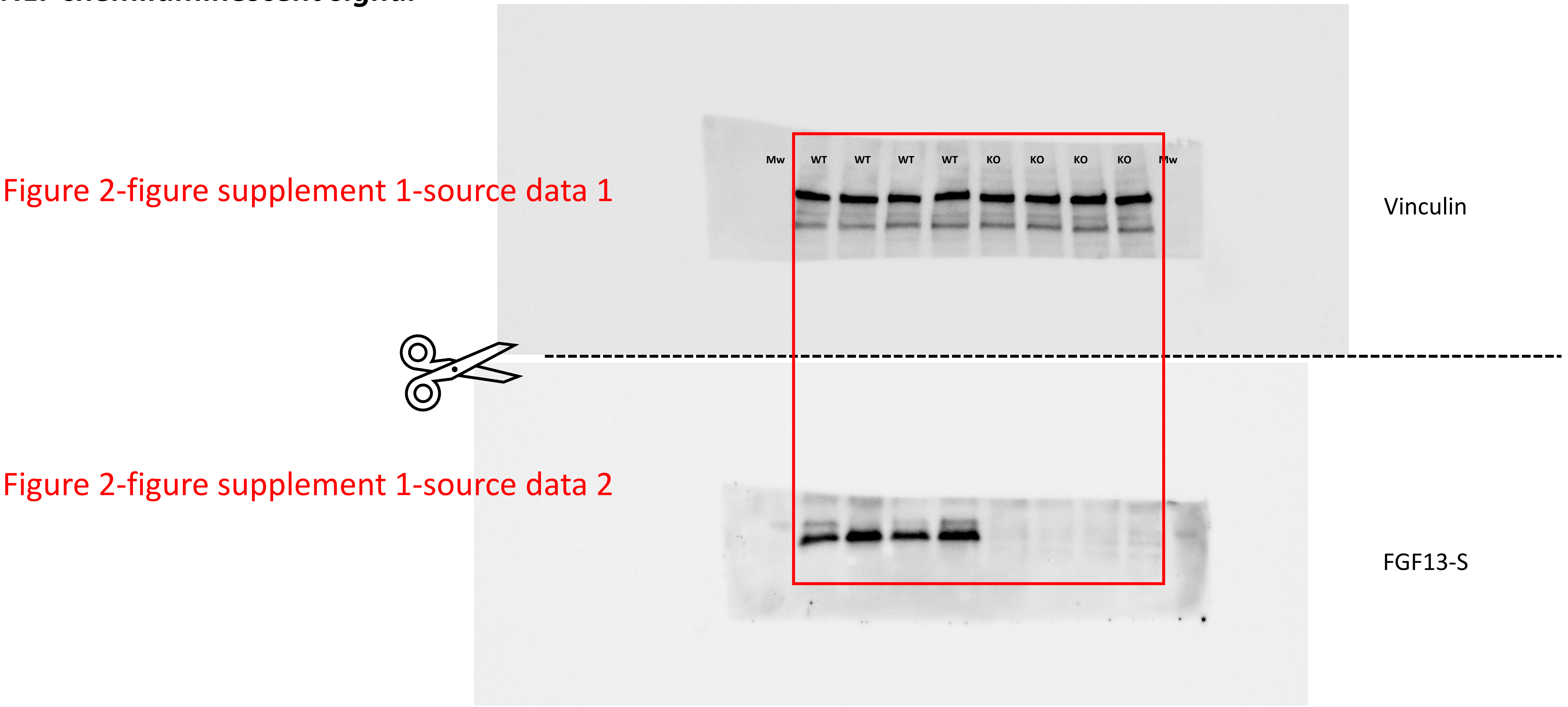

Gel cut where indicated, probed separately for vinculin (top) and FGF13 (bottom).
