# Supplementary material for: Interneuron FGF13 regulates seizure susceptibility via a sodium channel-independent mechanism
Source: eLife. 2025 Jan 8;13:RP98661. doi: 10.7554/eLife.98661 (PMC11709433; doi:10.7554/eLife.98661)
Supplement: Figure 3—source data 1. [file elife-98661-fig3-data1.zip › Figure 3C source data/Figure 3C-Source Data.pdf]

Figure 3 (panel C)-Source Data

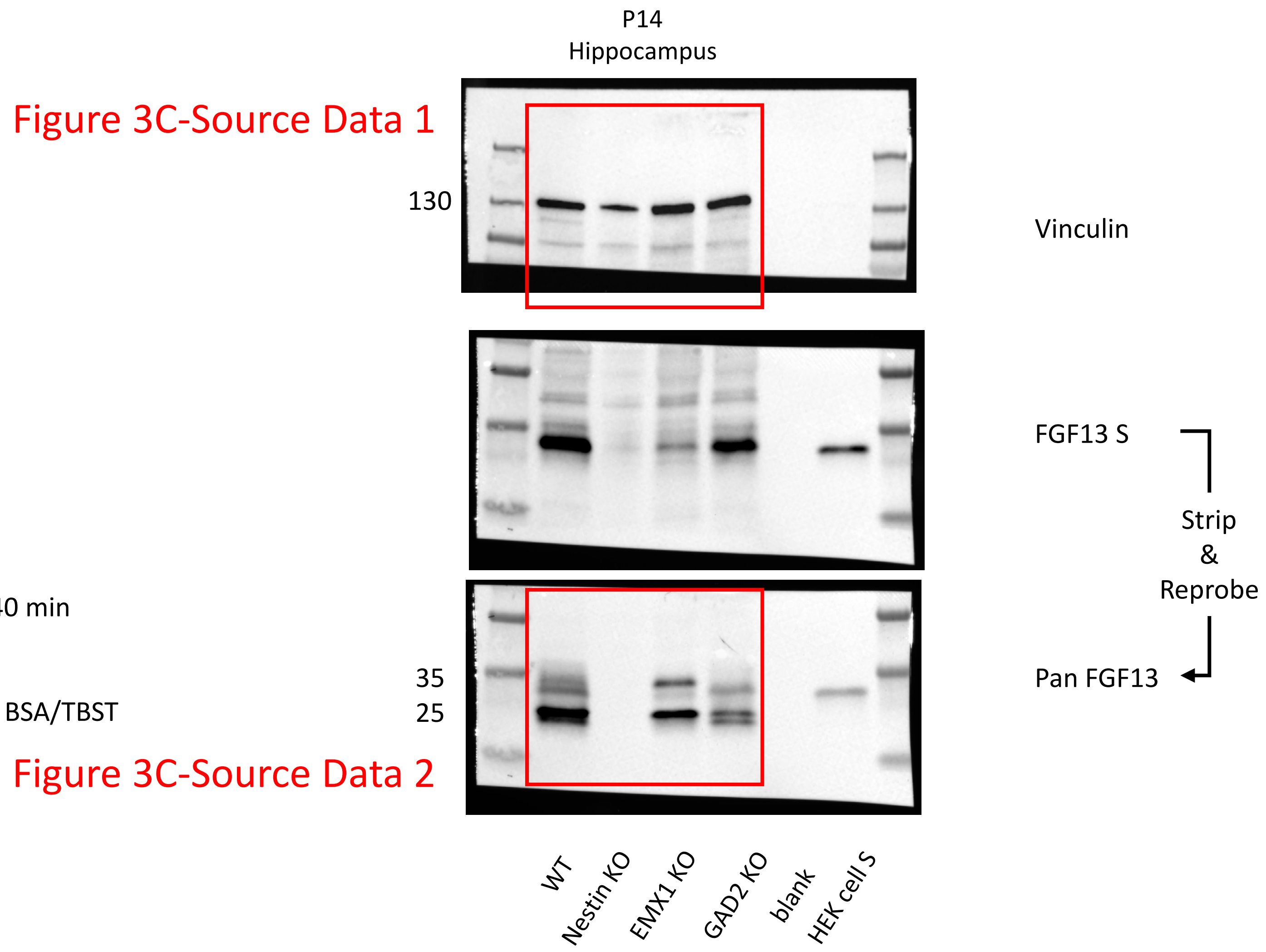

Imaged 4/25/23 & 4/27/23
Biorad 4-15% gel run 200V for 40 min
10ug brain tissue lysate loaded

1o AB: Pan FGF13 1:1000 in 5% BSA/TBST
O/N @ 4oC

Gel cut where indicated, probed separately for vinculin (top) and FGF13-S (middle), then stripped, and probed for FGF13-pan (bottom).
