# Supplementary material for: Interneuron FGF13 regulates seizure susceptibility via a sodium channel-independent mechanism
Source: eLife. 2025 Jan 8;13:RP98661. doi: 10.7554/eLife.98661 (PMC11709433; doi:10.7554/eLife.98661)
Supplement: Supplementary file 1. — Forward and reverse primer pair sequences and, where relevant, the PrimerBank ID. [file elife-98661-supp1.docx]

| **Supplemental Table 1: Primers used for real time quantitative polymerase chain reaction** | | | |
| --- | --- | --- | --- |
| **Forward (5’-3’)** | **Reverse (5’-3’)** | **Gene** | **PrimerBank* ID** |
| TCAGAGGGAAGCACAGTAGAC | TTCCACGCTGATTTGACAGCA | *Scn1a* | 9055328a1 |
| TTCATGGCTTCCAATCCCTCC | GGTGTCACGTCAGTCTTCTCT | *Scn2a* | 26328015a1 |
| CAGACCATGTGCCTTATTGTGT | CCGCGATCTGGAGGTTGTT | *Scn3a* | 9055330a1 |
| ATGGGGTAGGCTCTCCGAG | CCGACTCTGACTTAAACACCTTC | *Scn8a* | 6755410a1 |
| GATCAAGATCATTGCTCCTCCTG | AGGGTGTAAAACGCAGCTCA | *Actb* |  |
| *References for PrimerBank:  Spandidos A, et al. A comprehensive collection of experimentally validated primers for polymerase chain reaction quantitation of murine transcript abundance. *BMC Genomics*. 2008;9:633.  Spandidos A, et al. PrimerBank: a resource of human and mouse PCR primer pairs for gene expression detection and quantification. *Nucleic Acids Res*. 2010;38(database issue):D792–D799 | | | |
